# Convulsions in Children

**Published:** 1891-07

**Authors:** 


### CONVULSIONS IN CHILDREN.

Fits occur at all ages. During childhood they are called convulsions, and may come on within a few hours of birth. When a baby has a convulsion he should be at once undressed and put into a bath of warm water. The water must feel comfortably warm to the hand ; in this the child should be kept until the seizure is over. If wind escapes from the bowels, an injection of warm water will cause an action of the bowels and often immediate relief. Fits occurring in babies are nearly always due to wrong food or overfeeding. The nervous system of babies is more developed than other organs, consequently any food that disagrees may cause a convulsion. To avoid these alarming attacks mothers must not overfeed their offspring nor give them wrong foods. A baby fed by the bottle or on the bosom does not need feeding more than once every four hours during the day, and once or twice in the night. Babies must not be given any foods but the bosom milk, or the milk of the cow, goat, or ass, until it has cut at least two teeth, and even then not before it is nine months old. To give bread and milk, or oatmeal, or biscuits, or arrowroot, or any patent food, is to render a baby liable to these attacks. When babies suffer from any kind of illness this often begins with a convulsion, as in measles, scarlatina, inflammation of the lungs, &c. When children from one to ten years old suffer from convulsions, it is usually because food or other irritant is disturbing the stomach or bowels. A child whose nervous system is highly developed may have a fit if he has eaten some food which has disagreed with it. In a case of this kind the clothes about the neck must be unfastened, a cork or bit of wood placed between the teeth to prevent the tongue from being bitten, and hot fomentations should be applied over the stomach and bowels. If the attack is due to indigestible food, making the child sick by giving salt and water, mustard and water, or the tickling the throat with a feather, will quickly stop the attack.

